# Transcriptome sequencing and comparative transcriptome analysis of the scleroglucan producer *Sclerotium rolfsii*

**DOI:** 10.1186/1471-2164-11-329

**Published:** 2010-05-26

**Authors:** Jochen Schmid, Dirk Müller-Hagen, Thomas Bekel, Laura Funk, Ulf Stahl, Volker Sieber, Vera Meyer

**Affiliations:** 1 Chair of Chemistry of Biogenic Resources, Straubing Centre of Science, Technische Universität München, Schulgasse 16, 94315 Straubing, Germany; 2 Department of Microbiology and Genetics, Berlin University of Technology, Gustav-Meyer-Allee 25, 13355 Berlin, Germany; 3 Computational Genomics, Center for Biotechnology (CeBiTec), Bielefeld University, D-33594 Bielefeld, Germany; 4 Degussa Food Ingredients GmbH, Lise Meitner Str. 34, 85354 Freising, Germany; 5 Molecular Microbiology and Biotechnology, Institute of Biology, Leiden University, 2333 BE Leiden, The Netherlands; 6 PolyPhag GmbH, Robert-Rössle-Str. 10, 13125 Berlin, Germany

## Abstract

**Background:**

The plant pathogenic basidiomycete *Sclerotium rolfsii *produces the industrially exploited exopolysaccharide scleroglucan, a polymer that consists of (1 → 3)-β-linked glucose with a (1 → 6)-β-glycosyl branch on every third unit. Although the physicochemical properties of scleroglucan are well understood, almost nothing is known about the genetics of scleroglucan biosynthesis. Similarly, the biosynthetic pathway of oxalate, the main by-product during scleroglucan production, has not been elucidated yet. In order to provide a basis for genetic and metabolic engineering approaches, we studied scleroglucan and oxalate biosynthesis in *S. rolfsii *using different transcriptomic approaches.

**Results:**

Two *S. rolfsii *transcriptomes obtained from scleroglucan-producing and scleroglucan-nonproducing conditions were pooled and sequenced using the 454 pyrosequencing technique yielding ~350,000 reads. These could be assembled into 21,937 contigs and 171,833 singletons, for which 6,951 had significant matches in public protein data bases. Sequence data were used to obtain first insights into the genomics of scleroglucan and oxalate production and to predict putative proteins involved in the synthesis of both metabolites. Using comparative transcriptomics, namely Agilent microarray hybridization and suppression subtractive hybridization, we identified ~800 unigenes which are differently expressed under scleroglucan-producing and non-producing conditions. From these, candidate genes were identified which could represent potential leads for targeted modification of the *S. rolfsii *metabolism for increased scleroglucan yields.

**Conclusions:**

The results presented in this paper provide for the first time genomic and transcriptomic data about *S. rolfsii *and demonstrate the power and usefulness of combined transcriptome sequencing and comparative microarray analysis. The data obtained allowed us to predict the biosynthetic pathways of scleroglucan and oxalate synthesis and to identify important genes putatively involved in determining scleroglucan yields. Moreover, our data establish the first sequence database for *S. rolfsii*, which allows research into other biological processes of *S. rolfsii*, such as host-pathogen interaction.

## Background

The basidiomycete *Sclerotium rolfsii *is a soilborne plant pathogenic fungus causing diseases in many agricultural and horticultural plants [1-3]. However, it is also used in biotechnology as a microbial platform for the production of the exopolysaccharide (EPS) scleroglucan. This polysaccharide is a water-soluble homopolymer composed of a (1 → 3)-β-linked glucopyranose backbone with single (1 → 6)-β-linked glucopyranosyl branches on every third subunit [4] and traded under the commercial names Tinocare^® ^GL and Actigum^®^. Scleroglucan shows remarkable rheological properties rendering the substance as a multipurpose compound for many industrial applications, ranging from oil recovery over food industry to cosmetics and medical applications [5-7]. Surprisingly, only very little information is available on the biosynthesis of scleroglucan formation by *S. rolfsii *[4,7,8] whereas the physicochemical properties of scleroglucan are well explored [7-11].

According to theoretical considerations put forward by Sutherland [11,12], scleroglucan synthesis follows the general scheme for polysaccharide production in microbial systems in three major steps: substrate uptake, intracellular formation and extrusion from the cell. Uptake of glucose into the cell is mediated by glucose transporter(s), followed by phosphorylation of glucose to glucose-6-phosphate via a hexokinase reaction (EC: 2.7.1.1). After interconversion of glucose-6-phosphate to glucose-1-phosphate by phospho-glucomutase (EC: 2.7.5.1), a UTP-glucose-1-phosphate uridylyltransferase (EC: 2.7.7.9) activates glucose-1-phosphate to UDP-glucose. A (1 → 3)-β-glucan synthase (EC: 2.4.1.34) polymerizes the backbone chain using UDP-glucose as monomeric precursor. The last step yielding to the (1 → 6)-β branching at every third glucose molecule is supposed to be catalyzed by trans-D-glucosidases [12]. ^14^C incorporation experiments evidenced that the (1 → 3)-β chain of scleroglucan is elongated toward the non-reducing terminus and that (1 → 6)-β-linked glycosyl side residues are incorporated simultaneously as the (1 → 3)-β-glucan backbone is elongated [13,14].

Several empirical studies have been performed to identify optimum medium composition for EPS production by *S. rolfsii *[15-21]. Interestingly, medium conditions favoring scleroglucan production have been reported to increase the amount of secreted oxalate as well [22,23]. The biosynthesis of scleroglucan has thus been proposed to be closely linked to the synthesis of oxalate; a reducing agent and strong acid involved in the infection process of *S. rolfsii *[24,25]. During industrial scleroglucan production, however, the formation of the by-product oxalate is undesirable as it lowers the productivity of the process and negatively interferes with downstream processing of scleroglucan [7,18]. For some of its applications, e.g. in cosmetics and food industry, a cost intensive removal of oxalate is necessary.

Microbial oxalate is assumed to be synthesized in the glyoxylate cycle (GLOX), which is the anaplerotic pathway during growth on C2-carbon sources. Glyoxylate and succinate are the products of the isocitrate lyase reaction, and glyoxylate is either further oxidized to oxalate via the glyoxylate oxidase or used as precursor for malate synthesis. Although for basidiomycetes the cellular role of oxalate is still not clarified, it has been reported to be important for free radical formation, iron and calcium chelation as well as pectin and cellulose hydrolysis [26-29]. In phytopathogenic fungi, oxalate has been described as a very important factor contributing to fungal virulence. One role of oxalate is to lower the pH of the ambient environment, resulting in increased fungal polygalacturonase activity necessary for plant cell wall degradation [23,27,28]. Other roles include sequestration of calcium from cell walls, hydrolysis of plant pectin, suppression of plant defense responses and induction of the programmed cell death in plants [30-32].

Understanding the genetic basis for scleroglucan and oxalate biosynthesis is a prerequisite for the design of genetically engineered strains with improved scleroglucan yields. However, the genome of *S. rolfsii *has not been sequenced yet and DNA sequences have been published for only a few *S. rolfsii *genes. To overcome this obstacle, we applied the massively parallel short-read 454 pyrosequencing technology to sequence the transcriptome of *S. rolfsii*. From the assembled and annotated unigene sequences, we predicted genes particularly involved in EPS and oxalate metabolism. Additionally, we performed a global suppression subtractive hybridization (SSH) approach to isolate and identify genes up-regulated under scleroglucan-producing conditions. We used the sequence data obtained from the 454 sequencing and from the SSH approaches to finally develop Agilent microarray chips to perform comparative gene expression profiling for *S. rolfsii *grown in scleroglucan-producing and scleroglucan-nonproducing conditions and to identify genes differentially expressed under both conditions.

## Results and Discussion

### Designing scleroglucan-producing and scleroglucan-nonproducing media

**Figure 1:**
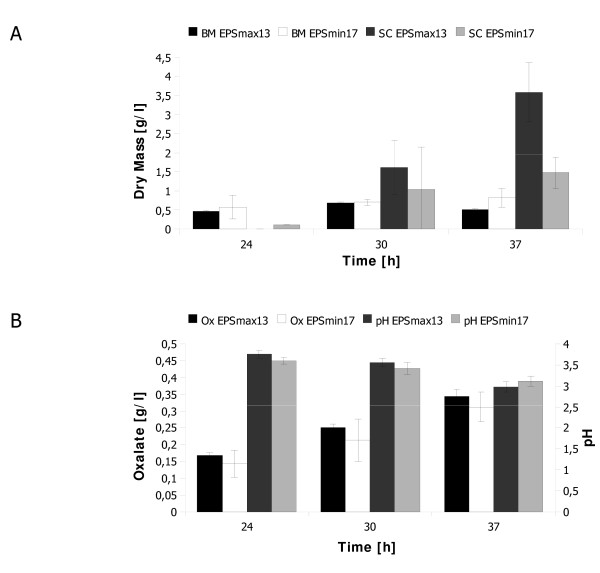
**Growth of *S. rolfsii *and metabolite production in EPSmax13 and EPSmin17 medium**. *S. rolfsii *was cultivated in 50 ml medium at 28°C up to 37 h. Cultures were harvested at the time points indicated, and the dry weight biomass (BM), scleroglucan (SC), oxalate and the medium pH determined. Mean values of a biological duplicate experiment are shown.

A basic requirement for this work was the development of two cultivation media for *S. rolfsii*, which should provide sufficient growth and a comparable biomass production, however with significant differences in EPS production. In order to identify such media compositions, we used the synthetic EPS medium proposed by Farina et al [15], and altered both the nature and concentration of the carbon (glucose, fructose, sucrose; 25-220 mM) and nitrogen (NH_4_Cl, NaNO_3_, (NH_4_)_2_SO_4_; 17-280 mM) sources. *S. rolfsii *was cultivated in these media and the formation of scleroglucan and oxalate was monitored over time (data not shown). As shown in Figure 1A, scleroglucan production was high in medium containing 220 mM Glc and 35 mM NaNO_3 _(designated EPSmax13) and lower in medium containing 220 mM Fru and 35 mM NH_4_Cl (designated EPSmin17). At 30 h of cultivation, *S. rolfsii *produced scleroglucan in EPSmax13 medium but to a slightly lesser extent in EPSmin17 medium. Sufficient amounts and significant differences in scleroglucan production are detectable after 37 h of cultivation, whereas biomass accumulation was comparable. We thus decided to choose the 37 h time point for the comparative analysis. Interestingly, cultures of *S. rolfsii *grown in EPSmax13 and EPSmin17 media displayed similar pH and oxalate profiles, suggesting that oxalate production is rather coupled to growth and biomass formation than to scleroglucan synthesis (Figure 1B).

### 454 pyrosequencing and data analysis

Total RNA extracted from 37 h old cultures of *S. rolfsii *grown in EPSmin17 and EPSmax13 medium were pooled in a 1:1 ratio to guarantee equal predominance of both RNA populations and subsequently reversed transcribed into cDNA. The mixed cDNA sample was sequenced by 454 Life Sciences™. The rationale behind combining both mRNA populations was to increase transcriptome coverage. Triplicate sequencing runs resulted in 356,098 single reads composed of 3.68 million bases (Table 1). Using the 454 Life Sciences™ Newbler software, these reads were trimmed and assembled into 21,937 contiguous sequences and 171,833 singletons (Table 1, Additional file 1) and are later on referred to as unigenes. A complete list of all unigenes has been deposited at the NCBI Sequence Read archive (SRA, http://www.ncbi.nlm.nih.gov/sra) under accession number SRA012273.1.

**Table 1:** Sequencing, assembly and data analysis.

Sequencing reads	356,098
Trimmed reads	343,410
Singletons	171,833
Average length singletons (bases)	77
Contigs	21,937
Largest contig size (bases)	1,256
Average large contig size (bases)	654
No. of bases in large contigs	286,124
No. of large contigs	437
No. of bases	3,681,160 bases

All unigenes obtained were functionally analyzed via the Sequence Analysis and Management System (SAMS). This software platform was originally developed to support the computational analysis of shotgun genome sequencing projects [33]. However, in addition to quality assessments, SAMS is well suited for the annotation of short sequence fragments and as an annotation pipeline also includes standard bioinformatics tools such as BLAST [34]. We thus used SAMS to analyze and functionally annotate the *S. rolfsii *unigenes. The analysis pipeline was set up with different BLAST tools and databases: BLAST2× versus the NCBI NR protein database (E-value cut-off of 10^-5^), BLAST2× versus the KOG protein database (E-value cut-off of 10^-5^), BLAST2n versus the NCBI NT nucleotide database (E-value cut-off of 10^-5^) and TBLASTx2 versus the NCBI NR/NT database, E-value cut-off of 10^-5^). The EuKaryotic Orthologous Groups database (KOG) is essentially the eukaryotic version of the Clusters of Orthologous Groups database (COG; http://www.ncbi.nlm.nih.gov/COG/).

**Figure 2:**
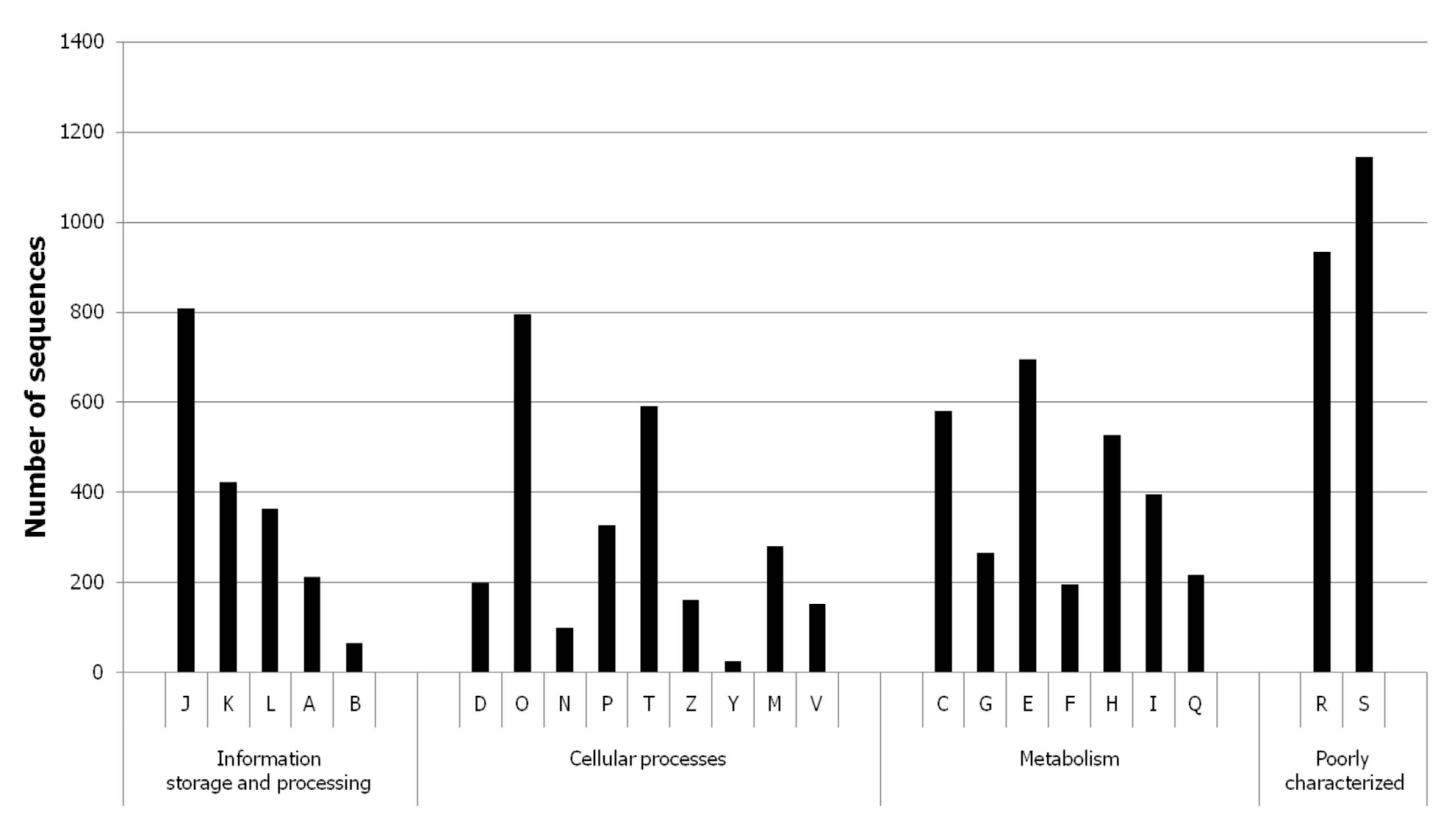
**KOG categorization of *S. rolfsii *unigenes**. Categories are abbreviated as follows: J, translation, ribosomal structure and biogenesis; K, transcription; L, replication, recombination and repair; A, RNA processing and modification; B, chromatin structure and dynamics; D, cell cycle control, cell division, chromosome partitioning; O, posttranslational modification, protein turnover, chaperones; N, cell motility; P, inorganic ion transport and metabolism; T, signal transduction mechanisms; Z, Cytoskeleton; Y, Nuclear structure, M, cell wall/membrane/envelope biogenesis; V, defense mechanisms; C, energy production and conversion; G, carbohydrate transport and metabolism; E, amino acid transport and metabolism; F, nucleotide transport and metabolism; H, coenzyme transport and metabolism; I, lipid transport and metabolism; Q, secondary metabolites biosynthesis, transport and catabolism; R, general function prediction only; S, function unknown.

**Figure 3:**
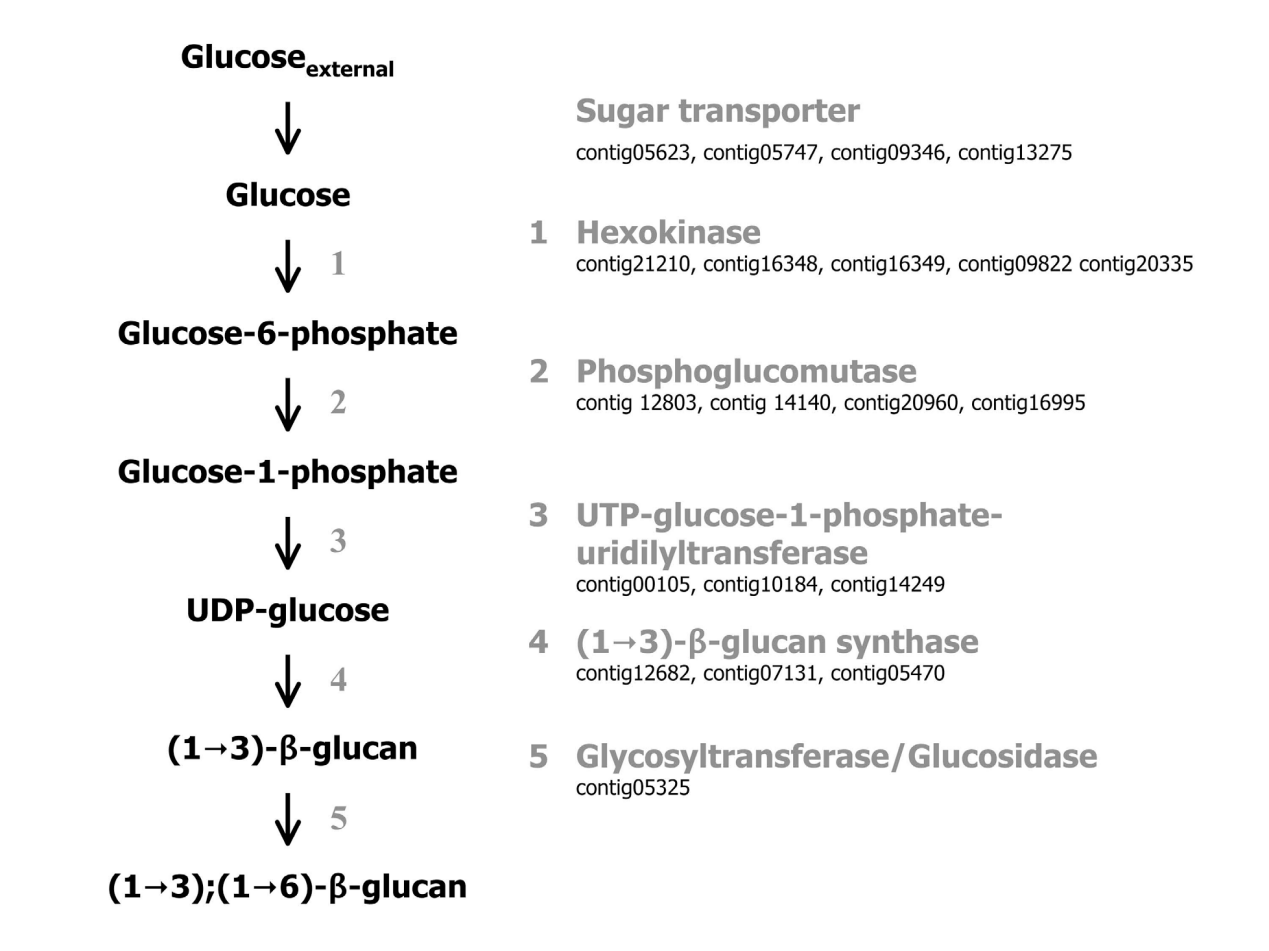
**Candidate unigenes potentially involved in scleroglucan biosynthesis**. Unigenes with predicted functions in one of these steps are indicated with their contig code.

A total of 6,951 sequences were assigned to one or more KOG functional categories. The remaining sequences were excluded by the chosen cut-off E value of 10^-5^. To evaluate the completeness of the transcriptomic data collection, we searched the unigenes for the presence of genes predicted to function in four primary carbon metabolic pathways - glycolysis, pentose phosphate pathway, TCA and glyconeogenesis. Annotated sequences were found for every step of the four pathways (data not shown), suggesting that the transcriptomic library could represent a nearly complete sequence database for the *S. rolfsii *transcriptome. The annotated unigene functions cover a broad range of KOG categories (Figure 2, Additional file 2), with the majority of genes grouping into the metabolism category. Among the functional KOG categories, we were particularly interested in the categories 'Carbohydrate transport and metabolism (G)' and 'Energy production and conversion (C)' as they were supposed to contain unigenes which participate in scleroglucan and oxalate metabolism. An overview of all unigenes allocated into both categories is given in Additional file 3. From this list, unigenes were selected which could potentially be involved in each of the five steps of scleroglucan biosynthesis (Figure 3). Surprisingly, only one potential candidate glycosyltransferase, presumed to catalyze the last step in scleroglucan synthesis, was identified. Lacking more direct hits, we screened the complete 171,833 singletons for the presence of a predicted glycosyltransferase unigene and retrieved one additional positive hit (D6LAZMP02HU01 M, 109 bases).

**Figure 4:**
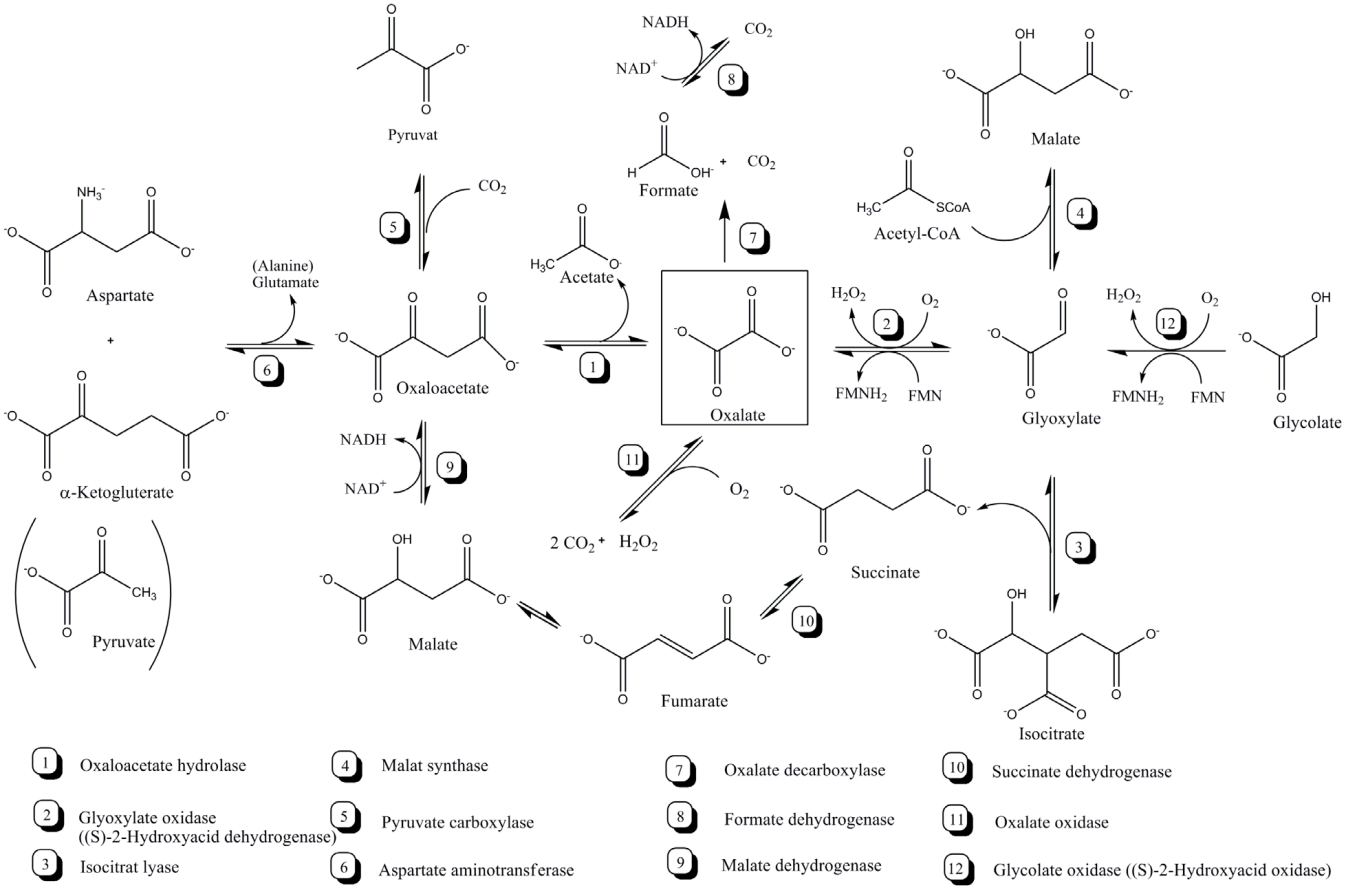
**Biochemical pathways involved in microbial oxalate metabolism**. A literature survey of microbial and especially fungal oxalate metabolism was conducted to obtain an inventory of all possible metabolic routes for oxalate synthesis and degradation. Unigenes with predicted functions in one of these steps are indicated with their contig code in Table 2.

With respect to oxalate metabolism, we could retrieve matching unigenes for 9 out of 12 possible enzymatic reactions (Figure 4 and Table 2). As three hits potentially encode an oxaloacetate hydrolase (reaction 1 in Figure 4) but none a glyoxylate oxidase (reaction 2 in Figure 4), it can be suggested that the main route for oxalate synthesis in *S. rolfsii *is via oxaloacetate. This would be in good agreement with previous findings which demonstrated that the most important pathway leading to oxalate formation in asco- and basidiomycetes is catalyzed via an oxaloacetate hydrolase and thus solely depends on oxaloacetate as precursor and not on glyoxylate [35-37]. On the other hand, it has been reported for *S. rolfsii *that the enzyme glycolate oxidase (reaction 12 in Figure 4) also accepts glyoxylate as substrate and oxidizes it to oxalate [20,38]. Four contigs show considerable homology to glycolate oxidases (Table 2), which thus could be candidate genes for such an enzyme.

**Table 2:** Unigenes with predicted enzyme function related to oxalate metabolism.

**No**.	Enzyme	Contig
1	Oxaloacetate hydrolase	contig05630 contig03818 contig14763

2	Glyoxylate oxidase	No hit

3	Isocitrate lyase	contig14763 contig15770 contig18874 contig18218 contig00175 contig08937

4	Malate synthase	contig00888 contig05791

5	Pyruvate carboxylase	contig00420 contig21272

6	Aspartate aminotransferase	contig02946 contig08150 contig16197 contig17262 contig19058 contig19059 contig20005

7	Oxalate decarboxylase	No hit

8	Formate dehydrogenase	contig00580 contig04723 contig04947 contig07858 contig11312 contig13166 contig15432 contig15438 contig16572 contig16914 contig17132 contig17885 contig18254 contig21037

9	Malate dehydrogenase	contig02004 contig07487e contig12545 contig16174 contig18066 contig19518 contig21582

10	Succinate dehydrogenase	contig11748 contig12058 contig05741 contig07994 contig14088 contig04161 contig15005 contig20092 contig17475 contig19516 contig18382 contig20164 contig20896 contig19935 contig21237 contig16039 contig19560

11	Oxalate oxidase	No hit

12	Glycolate oxidase	contig15511 contig21032 contig08342 contig17818

→ Summary of the *S. rolfsii *contigs giving at least one hit when analyzed with one of the four BLAST tools (E-value cut-off of 10^-5^) to the enzymes catalyzing reactions 1-12 according to Figure 4.

In terms of oxalate degradation, no hits were identified for an oxalate oxidase (reaction 11 in Figure 4) and an oxalate decarboxylase (reaction 7 in Figure 4), but several unigenes matched a formate dehydrogenase (reaction 8 in Figure 4). We propose two possible explanations for this finding. Either the main pathway for oxalate degradation is still the oxalate decarboxylase -- formate dehydrogenase route but the oxalate decarboxylase gene was expressed on a very low level and therefore not found among the mRNA population(s) used for sequencing. Or *S. rolfsii *does not use the oxalate decarboxylase -- formate dehydrogenase pathway for oxalate degradation and the formate dehydrogenase enzyme rather has a function in anaerobic respiration as shown for *Fusarium oxysporum *[39,40].

As the lack of detection for unigenes encoding for an oxalate oxidase and oxalate decarboxylase could be due to their low expression levels, we screened the genomic DNA of *S. rolfsii *via PCR using primers designed from respective fungal and plant gene sequences (see Methods). Basically, either one of both enzymes have been reported to be present in basidiomycetes, e.g. an oxalate oxidase is crucial for lignin degradation by the white rot fungus *Ceriopsis subvermispora *[41] and a oxalate decarboxylase is important for the brown rot fungus *Flammulina velutipes *for the survival under low external pH conditions [42]. All our PCR attempts to isolate a DNA sequence encoding an oxalate degrading enzyme were only successful for an oxalate oxidase but not for an oxalate decarboxylase (data not shown). We were able to isolate one DNA fragment (designated *oxox*), which showed 32% similarity to the barley *oxoX *gene, suggesting that the oxalate oxidase reaction is the likely oxalate degradation route in *S. rolfsii*.

### Comparative transcriptomics using suppression subtractive hybridization

**Figure 5:**
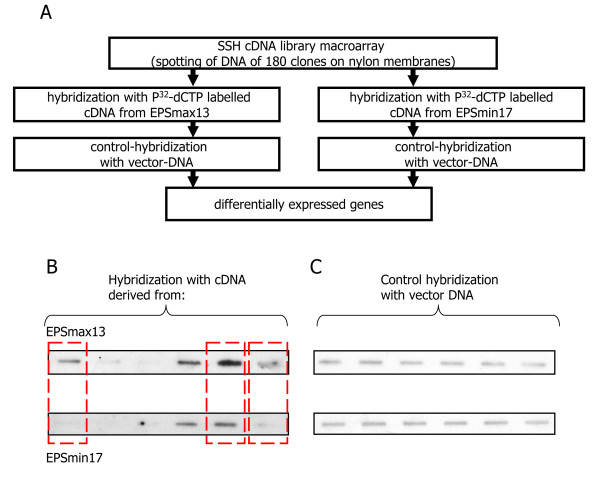
**Reverse Northern hybridization**. **A**, Flowchart of the Reverse Northern analysis. **B**, cDNA clones enriched in EPSmax13 were spotted via slot blot technique on two nylon membranes and then hybridized with total cDNAs derived either from EPSmax13 (upper panel) or EPSmin17 (lower panel). **C**, as reference, the same membranes were hybridized with vector-DNA to normalize probe intensities. Differentially expressed genes are marked by a red frame.

We used a suppression subtractive hybridization (SSH) approach to isolate cDNA species which are only present or enriched in *S. rolfsii *when grown in EPSmax13 medium compared to EPSmin17 medium. The advantage of the SSH approach is that also low abundant mRNA species can be isolated. The mRNA isolated from *S. rolfsii *cultivated for 37 h in EPSmax13 medium was used as 'tester' and mRNA isolated from 37 h old *S. rolfsii *cultures cultivated in EPSmin17 medium served as 'driver'. A total of 400 transformants representing cDNAs induced under scleroglucan-producing conditions were isolated. 180 of these clones were randomly selected and screened by reverse Northern hybridization for differential expression (Figure 5 and data not shown). 49 of the 180 screened cDNA clones showed considerable differences when hybridized with total cDNAs from scleroglucan-producing and scleroglucan-nonproducing conditions, respectively, confirming that these genes are up-regulated during scleroglucan biosynthesis. The 49 differentially expressed cDNAs were sequenced (Additional file 4), analyzed via TBLASTx and assigned to their predicted functional activity within different biochemical pathways (Table 3). In addition, the BioEdit tool http://www.mbio.ncsu.edu/BioEdit/bioedit.html was applied to blast and align the SSH unigenes against the 21,937 contigs identified via 454 sequencing (E-value cut-off of 10^-5^). For the majority of the SSH unigenes, we could identify homologous 454 unigenes (Table 3).

**Table 3:** Unigenes identified via SSH and Reverse Northern hybridization that display increased expression in EPSmax13 medium.

Target ID	Predicted function (TBLASTx)	Length (bp)*	Homologous 454 unigene
**Carbohydrate metabolism**			
12 VII-3	Glucan phosphorylase	421	contig00741
			contig15192
B5	UTP-glucose-1-phosphate uridylyltransferase	429	contig14249
F4	UDP-glucose-4-epimerase	531	contig14591
			contig16714
			contig05705
			contig19066
D1	Glucosamine-6-phosphate isomerase	441	contig18828
			contig19082
E3	Beta-fructofuranosidase	419	contig14026
			contig07977
C2	Glycogen phosphorylase	470	contig13256
7 VI-14	Glycogen phosphorylase	421	contig00741
			contig15192
3 VI-7	Isocitrate dehydrogenase	546	contig15308
			contig19633
9 VI-19	Oxoglutarate dehydrogenase	325	No hit
33 XI-28	Pyruvate decarboxylase	318	contig19196
			contig19387
E4	Pyruvate decarboxylase	242	No hit
G2	Pyruvate decarboxylase	236	contig19196
			contig19387
G8	Pyruvate decarboxylase	242	contig03793
			contig15593
D2	Phosphopyruvate hydratase	183	No hit
B8	Trehalose phosphorylase	178	contig17258
			contig19103
F3	Mannitol-1-phosphate dehydrogenase	357	contig21872
			contig13513
			contig19371
4 VI-8	Formate dehydrogenase	186	contig08513
27 V-36	Formate dehydrogenase	486	contig04947
			contig11312
			contig16572
			contig17132
			contig13166

**Lipid metabolism**			
29 X-12	Acetyl-CoA hydrolase/transferase	911	contig16913
			contig18900
			contig13924
34 XI-34	Oleate 12-hydroxylase gene	337	contig00730
			contig19568
21 VIII-38	Multifunctional beta-oxidation protein	589	contig15214
			contig15066
			contig12394
8 VI-18	Acyl-CoA-Dehydrogenase	877	contig11819
			contig00728
			contig07196

**Transport**			
13 VII-5	Endoplasmic reticulum-derived transport	451	contig14573
			contig13652
A3	Copper transporter	481	contig15666
			contig14830
			contig02543
			contig18312

**Amino acid metabolism**			
22 VIII-45	Acetylornithine aminotransferase	146	No hit
6 VI-12	Acetylornithine aminotransferase	140	No hit
21 VIII-38	Aminotransferase	589	contig15214
			contig15066
			contig12394
A2	Aspartate aminotransferase	476	No hit
B7	Aspartate aminotransferase	538	contig16197
			contig17262
			contig08150

**Oxidative stress**			
D4	Manganese superoxide dismutase	229	contig14464

**Others**			
D3	Superfamily of calcium sensors and calcium signal modulators	351	contig18978
			contig15800
			contig15801
			contig16990
18 VII-50	ATP synthase vacuolar proton pump	358	contig05644
			contig12704
20 VIII-21	GAL4-like DNA-binding domain	340	contig04517
31 X-30	Plasma membrane H+ transporting ATPase	348	contig14871
B4	Intradiol dioxygenase	570	No hit

**Hypothetical**			
A4	hypothetical protein UM02463.1	352	contig16014
14 VII-6	XP_001828655.1 CC1G_10527	345	No hit
24 IV-17	XP_001873967.1	470	No hit
28 X-11	XP_001875220.1	624	contig16711
			contig08447
5 VI-11	XP_001873416.1	392	contig01594
			contig20088
			contig21715
			contig15050
B2	XP_001830146.1 CC1G_09306	540	contig20176
			contig17567
			contig17591
			contig21673
15 VII-9	Transcription factor	352	No hit
B6	No hit	590	contig02865
			contig11989
			contig00561
A8	No hit	193	No hit
A7	No hit	193	No hit
1 VI-4	No hit	207	contig14768
			contig20467
			contig09075
11 VII-2	No hit	241	contig15360
			contig15302
G7	No hit	537	contig21711
			contig12931
			contig16495
C6	No hit	602	contig02865
			contig11989
			contig00561
F8	No hit	367	contig16532
			contig19793
			contig01157

* The DNA sequences of all SSH unigenes are given in Additional file 4.

Interestingly, we isolated not only genes predicted to function in scleroglucan and oxalate metabolism (e.g. UTP-glucose-1-phosphate uridylyltransferase, two aspartate aminotransferases, and two formate dehydrogenases) but also genes known to play fundamental roles in primary metabolism. For example, pyruvate decarboxylase (marker enzyme for oxygen limitation), isocitrate dehydrogenase (key enzyme of TCA), oxoglutarate dehydrogenase (enzyme of TCA and key enzyme for ammonia assimilation), acyl-CoA-dehydrogenase (first and rate-limiting step of fatty acid oxidation) and glycogen phosphorylase (crucial for survival under low energy supply) were among the predicted proteins.

### Comparative transcriptomics using Agilent microarray hybridization

Complementary to the SSH approach; we performed gene expression profiling to identify genes up- and down-regulated during scleroglucan-producing conditions. In order to manufacture respective Agilent microarrays, ten different 60 bp long probes were designed (Additional file 5) and *in situ *synthesized for all of the 454 and SSH unigenes (~22,000). The specificity of the probes was analyzed in a test hybridization run using pooled cDNA populations from *S. rolfsii *cultivated for 37 h in EPSmax13 and EPSmin17 medium (data not shown). Based on the results, two probes per unigene were selected for the design of Agilent Multiplex 44K Arrays (Additional file 6). The arrays were hybridized with *S. rolfsii *cDNA, obtained from 37 h cultivations in EPSmax13 and EPSmin17 medium, respectively. Hybridizations were performed in triplicate using mRNA isolated from three independent cultures (biological triplicate, Additional files 7, 8, 9, 10, 11, 12 and 13). After normalization based on quantiles, hybridization clustering experiments were performed to control both experimental conditions. Based on this quality check, we had to exclude one of the triplicate samples from further analysis (EPSmin17 experiment, Sample B) as it did not cluster with the other two EPSmin17 samples (Additional file 14).

For the comparison of the EPSmax13 triplicate versus the EPSmin17 duplicate arrays, we used an arbitrary chosen fold change of 2 to define unigenes as differently expressed (Students t-test; p < 0.05). Applying this filter, expression of a total of 723 unigenes did significantly vary between both conditions, whereby 356 unigenes were up- and 367 down-regulated under EPSmax13 condition when compared to the EPSmin17 condition. A comprehensive list of all differentially expressed unigenes is depicted in the Additional file 15. As not all of the 723 unigenes displayed a KOG annotation, we manually re-annotated this gene list using TBLASTx or BLASTN (E-value cut-off of 10^-5^) and classified the predicted protein functions according to the Functional Catalogue (FunCat) [43]. We could thereby assign putative FunCats to 267 unigenes, out of which 138 were up-regulated and 129 down-regulated in *S. rolfsii *when cultivated in EPSmax13 medium (Additional file 15, Figure 6).

**Figure 6:**
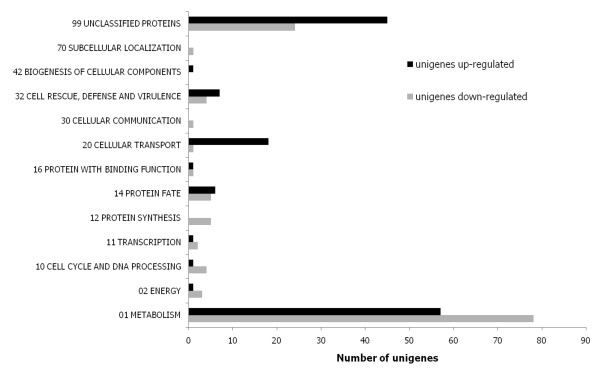
**Functional categories of genes up- or down-regulated in *S. rolfsii *grown in EPSmax13 medium compared to growth in EPSmin17 medium**. An annotated list of all responsive genes, including fold change, *p *value and classification, can be found in Additional file 15.

The functional categories with the largest number of differently expressed unigenes are the categories 'Metabolism' and 'Transport' (Figure 6). Among these are four unigenes which we had isolated via the SSH approach (e.g. glycogen phosphorylase, UDP-glucose-4-epimerase, formate dehydrogenase; Table 4). The high fold change cut-off used for microarray analysis as well as the lower sensitivity of microarrays compared to SSH probably limited the amount of overlapping hits. Nine unigenes predicted to encode polysaccharide-acting enzymes were up-regulated when *S. rolfsii *was cultivated in EPSmax13 medium (Table 4), thus representing potential candidate genes involved in scleroglucan elongation and branching. Moreover, many up-regulated unigenes fall into the group of ergosterol and sphingolipid metabolic proteins (Table 4). Finally, various unigenes assigned to transporters (ions, amino acids, peptides, lipids) and oxidoreductases (e.g. aryl-alcohol dehydrogenases) displayed altered expression under EPSmax13 conditions (Additional file 15). These transcriptional changes could imply that scleroglucan synthesis might be coupled to the cellular ion homeostasis machinery. Such a scenario would be in agreement with the overall concept that microbial EPS production is also an adaptive response towards environmental salt and osmotic stress [44-47].

**Table 4:** Unigenes selected from the microarray analysis that display increased or reduced expression in EPSmax13 medium compared to EPSmin17 medium.

Target ID	Predicted function (TBLASTx)	*P *value	Log_2_Fold
**Carbohydrate metabolism**			
contig13845	Glycogen debranching enzyme	6.43E-03	1.136
contig17335	Glycogen debranching enzyme	4.42E-02	1.174
contig18482	Glycogen debranching enzyme	8.15E-03	1.074
contig19066	UDP-glucose-4-epimerase	3.25E-03	1.012
contig20926	GH16 beta-1,3-glucan recognition protein	1.60E-02	1.542
contig01604	GH16 beta-1,3-glucan recognition protein	1.37E-02	1.540
C2	Glycogen phosphorylase	4.65E-02	1.493
contig04502	Glycoside hydrolase family 31	2.78E-02	1.455
contig08391	Glycoside hydrolase family 31	1.30E-02	1.102
contig20411	Glycoside hydrolase family 63	1.75E-03	1.056
F3	Mannitol-1-phosphate dehydrogenase	1.77E-03	1.305
contig07858	Formate dehydrogenase	1.97E-02	1.307
contig11312	Formate dehydrogenase	6.59E-04	2.341
contig13166	Formate dehydrogenase	1.15E-03	2.826
contig16572	Formate dehydrogenase	1.25E-03	2.611
contig16914	Formate dehydrogenase	6.76E-04	2.570
contig17132	Formate dehydrogenase	4.23E-02	2.360
contig21037	Formate dehydrogenase	1.59E-03	2.231
contig21586	Endocellulase	6.16E-03	-2.391
contig06887	Endocellulase	4.85E-02	-1.571
contig16192	Endocellulase	4.08E-03	-2.419
contig15791	Endocellulase	1.89E-02	-3.138
contig08327	Glucoamylase G2	1.39E-02	-1.069
contig04589	Glucoamylase G2	2.43E-03	-2.330
contig03614	Exo-beta-1,3-glucanase	7.25E-04	-2.473
contig10472	UDP-glucuronosyl/UDP-glucosyl transferase	3.21E-02	-1.403

**Lipid metabolism**			
contig04863	Squalene monooxygenase	4.27E-02	2.255
contig19483	Squalene monooxygenase	4.21E-02	2.007
contig18170	Squalene monooxygenase	4.40E-02	1.861
contig16238	Squalene monooxygenase	2.91E-02	1.546
contig16026	Sphingolipid hydroxylase	4.69E-03	1.858
contig01140	Sphingolipid hydroxylase	3.75E-02	1.741
contig08736	Sphingolipid hydroxylase	3.30E-02	1.625
contig19971	Sphingolipid hydroxylase	1.05E-02	1.610
contig03880	Sphingolipid hydroxylase	1.43E-02	1.291
contig11092	C-4 sterol methyl oxidase	2.49E-02	1.622
contig21591	C-4 sterol methyl oxidase	3.28E-02	1.524
contig03744	C-4 sterol methyl oxidase	4.50E-03	1.754
contig14837	C-5 sterol desaturase	7.16E-03	1.872

## Conclusions

In this study, we used different strategies to reveal genes involved in scleroglucan synthesis and oxalate metabolism of *Sclerotium rolfsii*, a fungus that lacks a sequenced genome. In sum, three independent transcriptomic approaches were applied, which together uncovered candidate genes for each predicted step of scleroglucan synthesis, oxalate synthesis and oxalate degradation. Many of these genes were unraveled in both global comparative transcriptomic analyses, making them as prime candidates for further analyses.

The insights into the genetics and transcriptome of scleroglucan synthesis obtained in this work are to our knowledge the first gained for any EPS produced by a basidiomycete. The sequence data covers a nearly complete set of genes transcribed in *S. rolfsii *and provides an important resource for studying the biology and pathogenesis of *S. rolfsii*.

## Methods

### Cultivation conditions

*S. rolfsii *strain ATCC15205 was cultivated at 28°C in shake flasks containing 50 ml EPS medium (C-source, N-source, 2 g/l K_2_HPO_4_, 0.5 g/l KCl, 0.5 g/l MgSO_4_*7H_2_O, 0.05 g/l FeSO_4_*7H_2_O, 1 g/l yeast extract, 0.7 g/l citric acid 7*H_2_O. pH 4.5) [15]. EPSmax13 contained 40 g/l glucose and 3.0 g/l NaNO_3 _as C- and N-sources, whereas EPSmin17 used 40 g/l fructose and 1.9 g/l NH_4_Cl, respectively.

### Analytical measurements

In order to determine *S. rolfsii *biomass from liquid cultures, 40 g of each culture broth were sampled, preheated to 56°C and subjected to enzymatic cell wall degradation (1 mg Glucanex/g broth). After incubation for 30 min at 56°C, Glucanex was heat-inactivated (90°C, 20 min) and the sample cooled down to room temperature. The initial weight (40 g) was re-adjusted by adding water and 30 g of this solution were centrifuged to harvest the biomass. The dry weight was determined after the wet biomass pellet was vacuum-dried over night (12 h, 60°C).

Scleroglucan levels were determined using isopropanol precipitation. Two volumes of isopropanol were mixed with one volume of culture broth and the resulting scleroglucan precipitate was filtered over a 74 μm mesh filter. After evaporation of isopropanol, the precipitate was vacuum-dried for 2 h at 60°C and the dry weight of scleroglucan determined.

Oxalate levels in the culture supernatant were determined via HPLC (Knaur column H+) using 0.05 M H_2_SO_4 _as solvent and an UV detector (210 nm).

### RNA isolation

Due to the high amounts of EPS produced, extraction of intact total RNA from *S. rolfsii *cultures was only possible by using a caesium chloride-based ultracentrifugation method [48]. In brief, 1 g of *S. rolfsii *mycelium was harvested by filtration and frozen in liquid nitrogen. After homogenization using a dismembrator (Braun Biotech), the pulverized homogenate was resuspended in 5 ml RNA extraction buffer (4 M guanidine isothiocyanate; 0.1 M Tris/HCl, pH 7.5; 1% β-Mercaptoethanol, 0.5% N-laurylsarcosine). After centrifugation (5000 ×g, 10 min, RT), the supernatant was subjected to ultracentrifugation using 5 M caesium chloride (30,000 ×g, 19 h, RT). The resulting RNA pellet was precipitated using 2 volumes of ice-cold EtOH (96%) and 1/10 volumes of 8 M LiCl.

### Suppression Subtractive Hybridization and Reverse Northern analysis

Suppression subtractive hybridization was performed using the PCR-SelectTM cDNA subtraction kit and followed the manufacturer's instructions (Clontech). *S. rolfsii *mRNA extracted from EPSmax13 cultures was used as tester (mRNA population containing specifically expressed transcripts) and mRNA isolated from EPSmin17 as driver (mRNA population that is used for subtraction). The tester cDNAs enriched under EPSmax13 conditions were ligated into pUC18 vector (Fermentas) and transformed into *Escherichia coli *DH5α (Gibco). Selected transformants were subjected to Reverse Northern analysis. Plasmid DNAs isolated from 180 randomly picked clones were slot-blotted onto positively Hybond-N nylon membranes (Amersham) and subjected to three independent hybridization runs using P^32^-labelled cDNAs generated from EPSmax13 and EPSmin17, respectively, as well as pUC18 plasmid DNA as probes. cDNAs were generated using Superscript II reverse transcriptase (Ambion). Hybridizations were performed using the Rapid-Hyb buffer system (Amersham) and followed the manufacturer's instructions.

### 454 pyrosequencing

Mixed cDNA populations obtained from *S. rolfsii *were sequenced in triplicate runs by 454 Life Sciences (Branford, USA). For this purpose, total RNA was isolated from 37 h old cultures of *S. rolfsii *grown in EPSmin17 and EPSmax13 medium (see above). Both RNA populations were pooled in a 1:1 ratio to guarantee equal occurrence and putative constitutively expressed genes (glycerol phosphate dehydrogenase, *gpdS*; glucoamylase G2, accession number D49448) were used for normalization. cDNAs were synthesized using the Clontech's SMART System protocol modified by AGOWA (Berlin, Germany). The cDNA library was sequenced by the ultrafast pyrosequencing method (454 Life Sciences).

### PCR screening

Oxalate oxidase metabolizes oxalate directly to CO_2 _and H_2_O_2 _(enzyme no. 11 in Figure 4) and is found mainly in plants [49-51] but also in basidiomycetes [41]. Sequences from barley (*oxoX*, CAA74595) and the fungus *Ceriopsis subvermispora *(CAD91553) were used to identify regions of high homology (data not shown), inside of which primers were designed (Bar 1, GGTACGAACACGTGGGC; Bar2, CCGGCCTCCACCCGAAGAG) to amplify a potential oxalate oxidase from *S. rolfsii *genomic DNA (see below). Using this primer pair, a ~850 bp fragment was isolated.

Oxalate decarboxylase degrades oxalate to formate and CO_2 _(enzyme no. 7 in Figure 4). Oxalate decarboxylases are present in the brown rot fungi *Postia placenta *[52] and *Flammulina velutipes *[42]. A region within the *F. velutipes oxdc *gene (AF200683), which is highly conserved among oxalate decarboxylases, was used as a template for the design of specific primers (Oxdc1, ATTAAGGATCCATCCATCGCATTTCCGATG; Oxdc2, AATACCDAYGTAGGAAATCATATCCGGCCG). For both PCR reactions, different annealing temperatures and elongation times were tested (not shown).

### Genomic DNA extraction

*S. rolfsii *was cultivated in 100 ml EPSmin17 medium at 28°C, 250 rpm using magnetic stirrers. After 48 h of cultivation, mycelium was harvested by filtration through a piece of gauze and washed twice with hot water (85°C) to remove scleroglucan. The mycelium was frozen in liquid nitrogen and genomic DNA extracted following a protocol described for *Aspergillus nidulans *[53].

### Microarray analysis

Tailor-made microarrays (44K multiplex chip, Agilent) were designed by imaGenes (Berlin, Germany) using an in-house developed method for empirical selection of best performing probes for each gene (Pre Selection Strategy). Briefly, up to ten probes were designed for each of the 454 and SSH unigenes as well as for the *oxox *gene (60 bp long oligomers). The 244K Agilent test array was hybridized with pooled Cy3-labeled cRNAs gained form EPSmax13 and EPSmin17 cultures (see above) and (in average) two of the best performing oligos were selected for each unigene.

For comparative expression profiling, total RNA was isolated from *S. rolfsii*, cultured for 37 h in EPSmax13 and EPSmin17 media as described above. RNA quality control, synthesis of Cy3-labeled cRNA including cRNA purification and cRNA quality control, microarray hybridization, scanning and data extraction (Agilent's feature extraction software) were performed by imaGenes GmbH. The complete set of transcriptional raw data is available as Additional files 8, 9, 10, 11, 12 and 13 and has additionally been archived at Gene Expression Omnibus http://www.ncbi.nlm.nih.gov/geo under accession number GSE21040. Expression data were analyzed by imaGenes GmbH using an in-house developed data analysis pipeline. After quantile normalization, genes were defined as differentially expressed if their expression levels varied at least 2 fold in EPSmax13 samples compared to EPSmin17 samples and if the difference was statistically significant (Student's t-test, *P*-value cut-off of 0.05).

## Authors' contributions

JS carried out the molecular genetic studies, sequence annotations and microarray analyses and performed the oxalate analyses. LF and JS carried out the SSH approach and extracted RNA and scleroglucan. TB participated in the bioinformatics and functional analyses. JS, DM and US participated in the design of the study. VM and VS conceived of, designed and coordinated the study. JS and VM wrote the manuscript. All authors read and approved the final manuscript.

## Supplementary Material

Additional file 1 **454 summary**. Summary of 454 sequencing and assembly. Click here for file

Additional file 2 **SAMS results**. Summary of annotation results using SAMS. Click here for file

Additional file 3 **KOG categories**. Unigenes grouped into the KOG categories 'Carbohydrate transport and metabolism (G)' and 'Energy production and conversion (C)'. Click here for file

Additional file 4 **SSH results**. Unigenes identified via SSH and Reverse Northern hybridization. Click here for file

Additional file 5 **60 mers**. Summary for all 60-mer probes designed for microarray hybridization. Click here for file

Additional file 6 **selected 60 mers**. 60-mer probes used for comparative microarray hybridization. Click here for file

Additional file 7 **Sample Key**. Sample key for comparative microarray hybridization. Click here for file

Additional file 8 **251706710004_2**. Raw data for experiment EPSmax13_37 h, Sample A. Click here for file

Additional file 9 **251706710005_4**. Raw data for experiment EPSmax13_37 h, Sample B. Click here for file

Additional file 10 **251706710005_1**. Raw data for experiment EPSmax13_37 h, Sample C. Click here for file

Additional file 11 **251706710005_3**. Raw data for experiment EPSmin17_37 h, Sample A. Click here for file

Additional file 12 **251706710004_4**. Raw data for experiment EPSmin17_37 h, Sample B. Click here for file

Additional file 13 **251706710006_4**. Raw data for experiment EPSmin17_37 h, Sample C. Click here for file

Additional file 14 **Dendrogram**. Dendrogram of the clustering hybridization experiment based on mean expression values. Click here for file

Additional file 15 **Differentially expressed unigenes**. Differentially expressed unigenes in EPSmax13 compared to EPSmin17 medium. Click here for file
